# Population Structure and Genetic Diversity of Tibetan Sheep Revealed by Whole-Genome Resequencing: Implications for Conservation and Breeding

**DOI:** 10.3390/genes16101232

**Published:** 2025-10-18

**Authors:** Junxia Zhang, Litan Zhang, Yuxiang Zhang, Yuting Deng, Xiaocheng Wen

**Affiliations:** College of Agriculture and Animal Husbandry, Qinghai University, Xining 810016, China

**Keywords:** population structure, genetic diversity, whole-genome resequencing, Tibetan sheep

## Abstract

Background: Tibetan sheep (*Ovis aries*) have evolved remarkable adaptations to the extreme high-altitude environment of the Qinghai–Tibet Plateau. While previous studies have identified some genetic features underlying these adaptations, a comprehensive understanding of their population genetics and selection signatures remains incomplete. We hypothesized that Tibetan sheep harbor unique genetic diversity and population structure distinct from low-altitude sheep (Hu sheep and Small Tail Han sheep), and that whole-genome resequencing could identify key positively selected genes driving their high-altitude adaptation and economic trait variation. Thus, this study aimed to characterize the population structure and genetic diversity of Tibetan sheep via whole-genome resequencing and identify genomic regions and candidate genes under positive selection related to high-altitude adaptation and important economic traits (growth, meat quality, wool, reproduction). Results: Using whole-genome resequencing of 90 Tibetan sheep (ZY) compared to 90 Hu sheep (HY) and 90 Small Tail Han sheep (XWHY), we identified significantly higher genetic diversity in Tibetan sheep (Pn = 0.6399, PIC = 0.1731). Population structure analyses revealed distinct clustering of Tibetan sheep, with principal components explaining 20.69% (PCA1), 12.26% (PCA2), and 14.18% (PCA3) of genetic variation. Selective sweep analysis identified 713 genomic regions (containing 207 genes) under positive selection, including key hypoxia adaptation genes (*HDAC5*, *BMP2/BMPR1B*, *DUOX2*) and economic trait genes (*FGF9* for growth; *SLC27A2* for meat quality; *KRTAP* for wool; *IZUMO1R* for reproduction). Functional enrichment highlighted pathways in oxygen transport (EPO regulation), energy metabolism (fatty acid β-oxidation), and vascular remodeling (TGF-β signaling). Conclusions: Our study provides the most comprehensive genomic characterization of Tibetan sheep to date, revealing both their unique genetic diversity and molecular mechanisms of high-altitude adaptation. The identified candidate genes offer valuable targets for marker-assisted breeding to improve productivity while maintaining adaptive traits, supporting sustainable development of plateau animal husbandry.

## 1. Background

Tibetan sheep, as a unique sheep breed native to the Qinghai–Tibet Plateau—a high-altitude region spanning ~2.5 million km^2^, with an average elevation exceeding 4000 m, extreme cold (annual average temperature −4 to 8 °C), low oxygen (oxygen partial pressure ~60% of sea level), intense ultraviolet radiation, and a short growing season for forage—have evolved to thrive in this harsh environment [1]. As the dominant livestock species in pastoral areas of Qinghai, Tibet, southern Gansu, and western Sichuan (core distribution zones of the plateau), Tibetan sheep exhibit distinct phenotypic and production characteristics: adult rams weigh 45–60 kg and ewes 35–50 kg; they are seasonal breeders with moderate fertility (average litter size 1.0–1.2, rarely twinning); their wool is coarse and dual-purpose (wool + down) with a fiber diameter of 25–35 μm, used locally for making tents, blankets, and warm clothing; and they are primarily raised as dual-purpose (meat + wool) livestock, with their meat (rich in protein, low in fat) being a staple protein source for plateau herders, and their wool supporting traditional livelihoods. For thousands of years, Tibetan sheep have been integral to the plateau’s pastoral economy and cultural heritage, while also playing a key role in preventing grassland desertification and maintaining alpine meadow ecological balance [2].

In-depth research into the population structure and genetic diversity of Tibetan sheep is of great significance for their conservation and breeding. Accurately understanding the population structure of Tibetan sheep can clarify the genetic relationships and geographical distribution characteristics, providing a scientific basis for rationally planning conservation areas and formulating targeted protection strategies [3]. Studies on genetic diversity help prevent inbreeding and safeguard the genetic resources of Tibetan sheep. Meanwhile, mastering the status of genetic diversity can aid in identifying superior genes, offering abundant genetic material for the genetic improvement of Tibetan sheep. This enhances their production performance and stress resistance, promoting the sustainable development of the Tibetan sheep industry.

At present, certain achievements have been made in the research on the population structure and genetic diversity of Tibetan sheep. Traditional research methods mainly include morphological observation [4], blood protein polymorphism analysis [5], and microsatellite marker analysis [6]. Through these studies, it has been preliminarily revealed that there are certain genetic differences among different populations of Tibetan sheep, and it has been found that Tibetan sheep have formed unique genetic characteristics adapted to the plateau environment during long-term evolution. With the advancement of high-throughput sequencing, whole-genome resequencing has emerged as a powerful tool for dissecting Tibetan sheep genetics, yet key limitations persist in existing research. Prior whole-genome sequencing studies on Tibetan sheep have made three core contributions while leaving critical gaps: in terms of adaptation gene discovery, early studies (e.g., with 15 Tibetan sheep [7] and with 20 Tibetan sheep [8]) identified hypoxia-related genes like *EPAS1* and *EGLN1*, but their small sample sizes (<25 individuals per breed) hindered the detection of rare adaptive alleles (frequency < 5%)—a limitation that obscures the full spectrum of high-altitude adaptation mechanisms; for population structure analysis, a recent whole-genome sequencing study compared 30 Tibetan sheep with 30 low-altitude Han sheep but only used principal component analysis (PCA) and FST to characterize population structure [9], lacking complementary tools such as Admixture or linkage disequilibrium (LD) decay, which results in an incomplete understanding of genetic differentiation; regarding economic trait linkage, most prior whole-genome sequencing research focused exclusively on either adaptation or productivity [7,8] in isolation. Yet, these approaches have not been applied to Tibetan sheep. Collectively, existing whole-genome sequencing research on Tibetan sheep is constrained by small sample sizes (*n* < 30 per breed), limited analytical depth (with only a few population or selection metrics), and a lack of integration between adaptation and economic traits—gaps that our study is designed to address.

This study aims to conduct a comprehensive and in-depth analysis of Tibetan sheep through whole-genome resequencing technology, thereby more accurately deciphering their population structure and genetic diversity. Specific objectives include the following: comprehensively identifying genetic variations such as single-nucleotide polymorphisms (SNPs), insertions/deletions (InDels), and structural variations (SVs) in the Tibetan sheep genome; based on genetic variation data, using methods such as principal component analysis (PCA), population structure analysis (STRUCTURE), and phylogenetic tree construction to deeply dissect the population structure and genetic relationships of Tibetan sheep; and through selective sweep analysis and other approaches, mining candidate genes and genetic markers related to important traits of Tibetan sheep and providing a theoretical basis for the genetic improvement of Tibetan sheep.

## 2. Materials and Methods

### 2.1. Animal Ethics Approval and Sample Background

Blood samples were collected from sheep as approved by Qinghai University Animal Ethics Committee (2020/PJ202401-75). The experiment was conducted in accordance with the Chinese Code of Practice for the Care and Use of Animals for Scientific Purposes (1988). All 270 sheep (90 per breed) were healthy adult ewes aged 2–3 years. This age group was selected because it represents the stable adult stage (avoiding juvenile genetic instability or senescent phenotypic changes), and using only ewes eliminated sex-related genetic variation (e.g., Y-chromosome loci) that could confound population structure analysis. Within each breed, the ewes had consistent body weight ranges: Tibetan sheep (38–45 kg), Hu sheep (42–48 kg), and Small Tail Han sheep (48–55 kg). While absolute weights differed (reflecting inherent breed differences), the narrow weight range within each breed ensured no extreme phenotypic outliers (e.g., undernourished or obese individuals) that could affect genomic data quality.

Tibetan sheep (Gonghe County, Qinghai) were raised under traditional extensive pasture-based management—grazing freely on natural alpine meadows year-round, with no supplementary feeding. Hu sheep and Small Tail Han sheep (Minghe County) were raised under semi-intensive management—housed in open-sided sheds at night and grazing on the pastures during the day, with daily supplementary feeding of 0.5 kg/head of concentrated feed (corn–soybean meal mix).

Tibetan sheep (Gonghe County, Qinghai): Sampled from areas with an average altitude of 3200–3500 m—a representative mid-high altitude zone of the Qinghai–Tibet Plateau, characterized by low oxygen (oxygen partial pressure ~65% of sea level), annual average temperature ~2 °C, and strong ultraviolet radiation (UV-B intensity ~1.5 times that of low-altitude areas). Hu sheep and Small Tail Han sheep (Minghe County, Qinghai): Sampled from areas with an average altitude of 2200–2500 m—the eastern low-altitude fringe of the Qinghai–Tibet Plateau, with milder climate (annual average temperature ~6 °C), higher oxygen partial pressure (~85% of sea level), and weaker UV radiation. The altitude difference between the two sampling regions (≈1000 m) aligns with the core contrast between the Tibetan sheep’s native high-altitude habitat and the introduced low-altitude breeds’ adapted environment, ensuring our genetic comparison could effectively capture altitude-related selection signatures.

### 2.2. Sample Collection and Blood Genomic Extraction

Blood samples were collected uniformly in mid-July 2024. About 5 mL blood sample per sheep was collected into EDTA-anticoagulated tube and stored at −20 °C until DNA extraction. Genomic DNA was extracted from the blood samples using the magnetic bead method with CWE9600 Magbead Blood DNA Kit (Kangwei Century Biotechnology Co., Ltd., Taizhou, China) to perform DNA extraction. The quality and concentration of the extracted DNA were determined by agarose gel electrophoresis and a NanoDrop spectrophotometer. DNA samples with an OD260/280 ratio between 1.8 and 2.0 and a concentration above 30 ng/μL were used for library construction. The samples were then sent to Compass Agritechnology Co., Ltd. (Tianjin, China) for whole-genome resequencing.

### 2.3. Whole-Genome Resequencing

During the library construction process, genomic DNA with a total amount greater than 1 μg was randomly fragmented into approximately 300–350 bp fragments using the universal library construction kit (for MGI)-NadPrep^®^ DNA and the Covaris™ fragmentation instrument. After end repair, A-tailing, and adapter ligation, DNA fragments of approximately 300–350 bp were selected using NadPrep^®^ SP Beads. PCR amplification was then performed, followed by purification of the PCR products using NadPrep^®^ SP Beads. The sequencing library was ultimately obtained. After the library was constructed, an initial quantitative analysis was conducted using Qubit 2.0. Subsequently, the insert fragments of the library were detected using Bioanalyzer^®^ (Agilent, Santa Clara, CA, USA), and once the results met expectations, the library was then subjected to sequencing on the DNBSEQ-T7 platform; the PE150 sequencing protocol was selected for sequencing.

### 2.4. Data Analysis

Quality Control: To ensure the quality of information analysis, fastp was used to conduct a series of quality control (QC) on the raw reads. Remove reads with adapters; remove paired reads when the proportion of N in the sequencing read exceeds 1% of the total base number of the read; and remove paired reads when the number of low-quality (Q ≤ 5) bases in the sequencing read exceeds 50% of the total base number of the read.

Read Mapping: After data filtering, an index was built based on the provided reference genome. Then, Clean Reads were aligned to the reference genome using the BWA0.7.17 (men) software. The Clean Reads were sorted and indexed using samtools 1.7. The GATK 4.1.8.0 software’s built-in modules were used to remove duplicates from the Bam files. Based on the Bam files, the sequencing depth and genomic coverage of each sample were then statistically analyzed to prepare for subsequent variant detection.

Variant Calling: Single nucleotide polymorphism (SNP) mainly refers to the DNA sequence polymorphism caused by the variation in a single nucleotide at the genomic level, including single base transitions and transversions. HaplotypeCaller module of the software GATK (4.18) was used to perform variant detection on multiple samples with the processed alignment files. The detected variants were filtered using VariantFiltration, and the SNPs were annotated using ANNOVAR (v2016Feb01), which performs gene-based annotation, region-based annotations, filter-based annotation, and other functionalities annotations. To reduce the error rate of SNP detection, SNPs were filtered as follows: QD < 2.0, FS > 60.0, MQ < 40.0, SOR > 3.0, MQRankSum < −12.5, ReadPosRankSum < −8.0; then, SNPs were strictly filtered: SNP cluster filtering (no more than 2 SNPs within 5 bp); SNP filtering near Indel (SNPs within 5 bp of Indel are filtered out); and for sites with GQ (Genotype Quality) less than 20.0, the genotyping quality tag of the sample was marked as low GQ.

Population Structure Analysis: Principal component analysis (PCA) was carried out using PLINK software (2.00a3.3) to visualize the genetic relationships among samples. The population structure was further analyzed using STRUCTURE 2.3 software, with the number of assumed populations (K). The multiple K values were determined based on the given population information (typically, K equals the number of populations), and population structure analysis was conducted using the Admixture 1.3.0 software. The smaller the cv value was, the better the corresponding K value was, and thus the optimal number of clusters (K) was determined to be the number of populations. The genetic distance matrix of IBS was constructed using Plink (version 1.9), and based on this, a phylogenetic tree was constructed using the Neighbor-Joining (NJ) method. From the analysis results, we can roughly infer which samples have more similar genetic characteristics.

Linkage Disequilibrium Analysis: Linkage Disequilibrium (LD) refers to the non-random combination relationship between different loci’ alleles within a population. That is, when the probability of two alleles (A, B) located on the same chromosome existing simultaneously is greater than the probability of their simultaneous occurrence due to random distribution within the population, these two loci are said to be in LD state. Usually, it is represented by the values of D’ and r^2^. Generally speaking, in linkage disequilibrium analysis, the LD value of wild species is relatively low, while that of domesticated species will be larger due to the positive selection effect. This is because in a DNA sequence, there is a linkage relationship between loci. Different loci’ linkages form “haplotypes”, and with the accumulation of recombination, specific haplotypes will be weakened and gradually disappear. Due to the relationship between recombination rate and linkage distance, the intensity of linkage disequilibrium between loci and their surrounding loci continuously decreases as the distance increases. For a newly generated haplotype, since recombination has not had time to disrupt the linkage between loci, the distance of linkage disequilibrium between them is often relatively far. Under neutral conditions, if a haplotype is relatively new, its frequency is often low, while a haplotype with a higher frequency will need a long period of time to reach a relatively high frequency due to the influence of random drift. If a population undergoes positive selection, the surrounding loci linked to favorable loci will rapidly increase in frequency due to the hitchhiking effect. Therefore, the haplotype containing the favorable locus has a higher frequency and, due to the short time it has experienced, also has a longer LD influence range. The PopLDdecay software 3.41 was used to calculate the r^2^ values of linkage disequilibrium for multiple populations. The r^2^ values of SNPs within different distances were calculated, and the attenuation of population LD r^2^ values with the increase in the distance between loci was observed. Generally, the r^2^ values of wild species decay faster than those of local varieties and cultivated varieties.

Genetic Diversity Analysis: Genetic diversity analysis includes the analysis of the proportion of polymorphic markers in populations (P_N_), the expected heterozygosity (H_E_), the observed heterozygosity (H_O_), and nucleotide diversity (P_i_). H_O_ and H_E_ were analyzed using the methods proposed by Sun et al. [10] and Nei [11]. The Plink software was used to calculate the minimum allele frequency (MAF) for each locus; then, a self-written R script was used to calculate P_N_. P_i_ was calculated using VCFtools 0.1.16 to evaluate the genetic diversity within each population. Polymorphism information content (PIC) is a quantitative measure used to assess the degree of polymorphism in genetic markers, primarily employed to evaluate the effectiveness of genetic markers in linkage analysis. PIC is calculated using the following formula:

PIC = 1 − ∑(p_i_^2^) − ∑∑(2p_i_^2^p_j_^2^)

where p_i_ and p_j_ represent the frequencies of the i-th and j-th alleles, respectively.

Selection Signature Analysis: The fixation index (FST) and genetic diversity (θπ) have been proven to be an extremely effective method for detecting selection elimination regions. Specifically, when exploring functional areas closely related to the living environment, it often leads to strong population evolution. The two methods jointly screen for strong population evolution, facilitating the selection of target genes. Here, the results of the two analysis methods were combined and selected the positions that appeared simultaneously in the top (0.01%) of the FST sliding window analysis and the top (0.01%) of the θπ Ratio analysis. These positions were taken as candidate target sites. The reference genome information of the corresponding species was downloaded from the ENSEMBL planform. The ANNOVAR software was used to annotate the candidate target SNPs onto the corresponding genes. Then, the gene function enrichment analysis of the annotated genes was conducted based on the gene ontology (GO) and Kyoto Encyclopedia of Genes and Genomes (KEGG) databases.

## 3. Results

### 3.1. Genetic Diversity

After initial variant detection and filtering, we implemented quality filtering (to ensure SNP reliability) and functional prioritization (to identify biologically relevant SNPs) to identify high-confidence, functionally relevant SNPs for downstream analysis (e.g., population structure, selection signatures). A total of 51,378 SNPs were screened out (Table S1), including their genomic location, associated gene, variant type, and functional impact. These SNPs are linked to core traits: to high-altitude adaptation (e.g., *HDAC5*, *BMP2*) and economic traits (e.g., *SLC27A2* for meat quality, *KRTAP13-1* for wool).

Genetic diversity analysis includes the analysis of the proportion of polymorphic markers (P_n_) in the population, the expected heterozygosity (H_e_) of the population, the observed heterozygosity (H_O_) analysis, and nucleotide diversity (P_i_) and polymorphism information content (PIC). The results of genetic diversity analysis for each population are presented in Table 1. The results indicated that Tibetan sheep (ZY) had an average Pn of 0.6399, which was not significantly different from Small Tail Han sheep (XWHY, 0.5975) or Hu sheep (HY, 0.638) (*p* > 0.05), indicating similar levels of polymorphic loci across breeds. Tibetan sheep exhibited the lowest He (0.2489) and Pi (0.2533) among the three breeds. Specifically, He of ZY was 8.29% lower than XWHY (0.2714) and 4.56% lower than HY (0.2608), while Pi of ZY was 8.22% lower than XWHY (0.2760) and 4.60% lower than HY (0.2655) (*p* < 0.05 for all comparisons). The polymorphic information content (PIC) of SNP loci in Small Tail Han sheep, Hu sheep, and Tibetan sheep were 0.1661, 0.1725 and 0.1731, respectively, indicating low polymorphism (PIC < 0.25) in all three populations.

### 3.2. Population Structure

**Figure 1:**
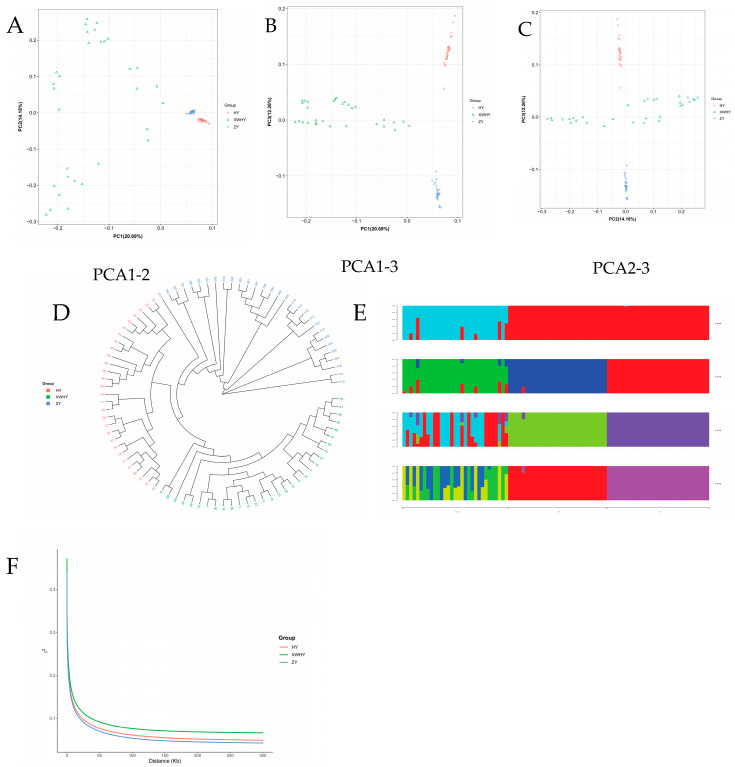
Principal component analysis (PCA) (**A**–**C**): The results based on filtered SNP markers. The PCA was conducted using filtered SNP markers to obtain the variance explained by each principal component (PC) and the score matrix of samples on each PC. The population structure was visualized through pairwise scatter plots (**A**–**C**) of the top three principal components. The colored shapes represent three populations: XWHY, HY, and ZY. The axes of the plots represent the scores of each PC, with the percentage values in parentheses indicating the variance explained by each PC, representing the proportion of total variance contributed by that PC in relation to all PCs. Phylogenetic tree analysis (**D**): The results showed the evolutionary relationships among different breeds. Evolutionary branches of species with closer genetic relationships often cluster together and are marked with the same color. Population structure analysis (**E**): The Admixture software was used to assess the goodness of fitting of the models with different numbers of populations (K) through cross-validation and outputted the corresponding cross-validation error (CV error). Generally, the smaller the CV value is, the better the model fits, and the corresponding K value is closer to the true situation. The optimal number of subgroups K for this analysis was 2. In a stacked bar plot, each bar represents an individual, and the bar was divided into several colored sections, with each color section indicating the proportion of genetic components from the corresponding ancestral population of that individual. Linkage disequilibrium analysis (**F**): The PopLDdecay software was used to calculate the r^2^ values of linkage disequilibrium for multiple populations and to determine the r^2^ values of SNPs within different distances. The vertical line represents the distance at which linkage disequilibrium occurs, while the horizontal axis represents the correlation coefficient of linkage disequilibrium.

Principal component analysis (PCA) revealed that the first three principal components (PCA1, PCA2, and PCA3) could explain for 20.69%, 12.26%, and 14.18% of the genetic variation, respectively (Figure 1A–C). Hu sheep and Tibetan sheep were relatively independent and clustered together, respectively. In contrast, Small Tail Han sheep exhibited more dispersed, indicating greater heterogeneity within this breed. This pattern suggests that after being introduced to the region, some Small Tail Han sheep individuals may have undergone hybridization, resulting in non-purebred descendants. A population phylogenetic tree was constructed by the Neighbor-Joining method using MEGA 12 software, and the result was shown in Figure 1D. This result was consistent with the PCA results. The three groups were clustered into groups, and the groups were relatively independent. The ZY clade was the most compact, reflecting low within-breed genetic diversity; the XWHY clade was the most divergent (consistent with PCA results); and the HY clade was intermediate in compactness. No inter-clade mixing was observed. Population structure analysis revealed that when K = 2, the population structure was best explained, with clear genetic admixture patterns among different populations. ZY and HY populations showed unique genetic components, while XWHY and the others had a degree of gene exchange (Figure 1E). The LD decay analysis Figure 1F) revealed the genetic diversity of different populations and the rate of decay for each population. Populations with rapid decay generally have higher genetic diversity, while those with slow decay generally have lower genetic diversity. In this study, the decay of ZY was relatively fast, indicating relatively higher genetic diversity, while the decay of HY was relatively slow, indicating relatively lower genetic diversity.

### 3.3. Selection Signature Analysis

**Figure 2:**
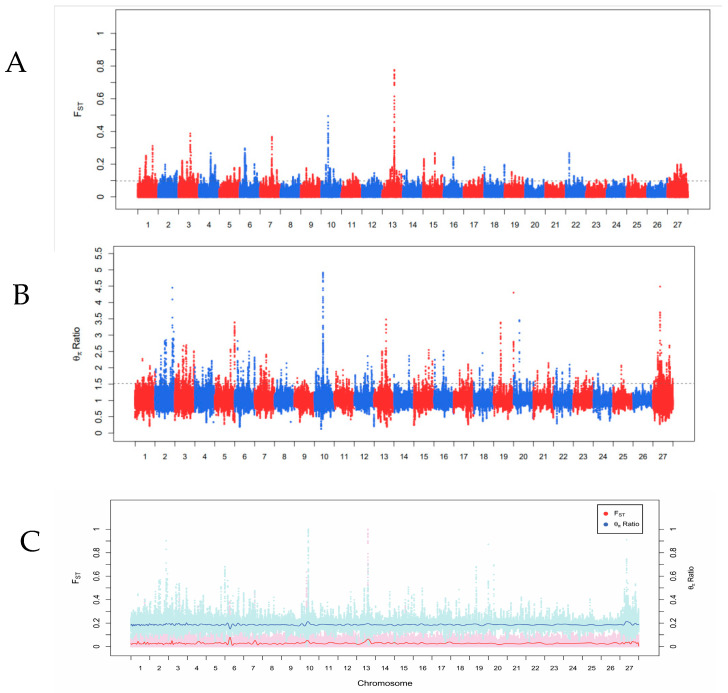

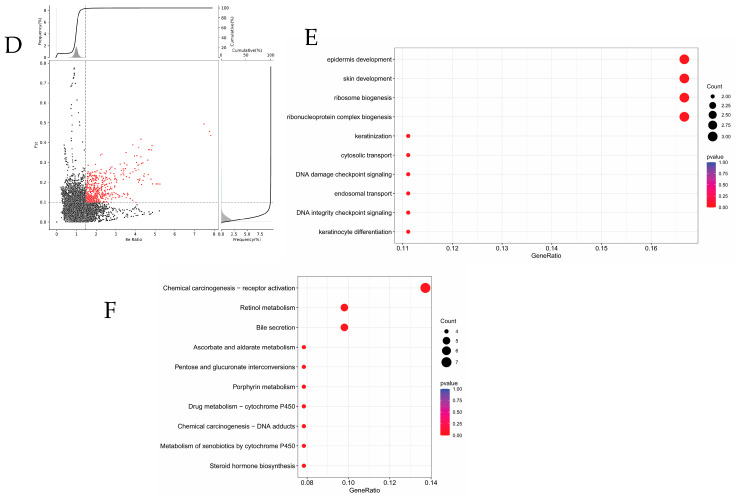
Visualization of the evolutionary analysis results of population ZY and population HY (**A**–**D**): The X axis represents the θπ Ratio, and the Y axis represents the Fst value, corresponding to the frequency distribution graphs on the top and right sides, respectively. The scatter plot in the middle represents the Fst and θπ Ratio values within different windows, among which the red points represent SNPs with both Fst and θπ Ratio values in the top 0.01%. Point plot of the biological process (BP) ontology enrichment analysis results of ZY population and HY population GO (**E**). Point plot of the KEGG pathway analysis results of ZY population and HY population KEGG (**F**).

A total of 713 genomic regions were identified as potential regions under positive selection. After annotating 713 candidate regions, a total of 207 genes were obtained (Table S2). Functional enrichment analysis of the genes located in these regions showed significant enrichment in several biological processes (Figure 2A–F), revealing significant enrichment in pathways such as ascorbate and aldarate metabolism, Pentose and glucoronate interconversions, retinol metabolism, chemical carcinogenesis—receptor activation, bile secretion, porphyrin metabolism, drug metabolism—cytochrome P450, chemical carcinogenesis—DNA adducts, metabolism of xenobiotics by cytochrome P450, steroid hormone biosynthesis, drug metabolism—other enzymes, oxytocin signaling pathway, cGMP-PKG signaling pathway, calcium signaling pathway, long-term potentiation, osteoclast differentiation, apelin signaling pathway, biosynthesis of cofactors, gap junction, cellular senescence, circadian rhythm, C-type lectin receptor signaling pathway, MAPK signaling pathway, type II diabetes mellitus, growth hormone synthesis, secretion and action, and oocyte meiosis. Functional enrichment analysis indicated that these candidate genes mainly play crucial roles in biological processes and cellular components. The enrichment results showed that the candidate genes are primarily involved in energy and antioxidation, blood vessels and circulation, hormones and growth, detoxification and metabolism, immunity and bone metabolism, and nervous system and reproduction. The genomic analysis revealed strong selection signals in key genes associated with hypoxia adaptation across multiple biological pathways. Within the hypoxia sensing and HIF signaling pathway, *HDAC5* was identified as stabilizing HIF-1α through deacetylation, thereby upregulating EPO and other target genes, while *BMP2* demonstrated angiogenic properties via VEGF pathway activation, enhancing skeletal muscle capillary density. *BMPR1B* emerged as a crucial mediator of BMP2 signaling, regulating pulmonary artery smooth muscle proliferation to mitigate high-altitude pulmonary hypertension. In the reprogramming of energy metabolism, *SLC25A46*, a mitochondrial carrier protein that enhances fatty acid β-oxidation efficiency under hypoxic conditions, regulates cellular metabolic adaptations. *NDUFS7*, a critical subunit of ETC Complex I, maintains ATP synthesis stability during oxygen deprivation, and *COQ5* is the coenzyme Q10 synthase that reduces ROS leakage while improving ETC efficiency. Vascular and hematological adaptation mechanisms were reflected in *LTBP2’s* regulation of TGF-β-mediated vascular remodeling to increase pulmonary artery elasticity, preventing high-altitude pulmonary hypertension, while *GJA3* maintained gap junction communication to coordinate cardiomyocyte electrophysiological synchronization under hypoxia, showing strong association with cardiac hypoxia tolerance. In the dual oxidase system, *DUOX2* played a pivotal role in balancing hypoxia-induced ROS production between signaling and oxidative damage. The oxidative stress defense system featured by the *DUOX2/DUOXA2* complex regulates H_2_O_2_ production as a key component of Tibetan sheep’s antioxidant capacity, alongside *SORD’s* maintenance of NAD^+^/NADH redox balance through its sorbitol dehydrogenase activity. Concurrently, the study identified multiple candidate genes linked to economically important traits. Growth-related genes included *BMP2* and *BMPR1B* regulating skeletal muscle differentiation and bone development, *FGF9* promoting muscle precursor cell proliferation, *IGF2BP2* stabilizing *IGF2* mRNA to enhance growth, and *MYCBP2* driving muscle fiber hypertrophy through cell cycle regulation. Meat quality traits were associated with *SLC27A2’s* modulation of intramuscular fat deposition, *LIPH’s* influence on flavor compound release through phospholipid metabolism, *PDGFD’s* promotion of adipocyte differentiation affecting subcutaneous fat thickness, and *HMBS*’s role in meat color stability via heme synthesis. Wool production characteristics involved KRTAP family genes determining fiber strength and fineness, *LTBP2* affecting wool crimp and elasticity through collagen regulation, and *GJA3* participating in follicular cell communication during fiber growth cycles. Reproductive performance was linked to *BMPR1B’s* effect on ovarian granulosa cell function through the *FecB* mutation, the *HOXA* family’s (particularly *HOXA10*) regulation of uterine receptivity, *IZUMO1R’s* mediation of sperm–egg binding, and *CYP19’s* control of estrous cycle regularity via estrogen synthesis catalysis. These candidate genes involved in the signaling pathways collectively form the comprehensive adaptation mechanism and production performance of Tibetan sheep in extreme environments such as high-altitude hypoxia, intense ultraviolet radiation, and cold conditions.

## 4. Discussion

Our study advances prior sheep whole-genome resequencing research—especially on Tibetan sheep—via three key, interrelated improvements. First, with 90 individuals per breed (total *n* = 270, a 3–6× increase over prior *n* = 15–30 per breed), we enhance statistical power to detect rare adaptive alleles (e.g., a *HDAC5* SNP undetectable earlier) and reduce bias in genetic diversity estimates. Second, we integrate six complementary analytical tools: for population structure, PCA + Admixture + phylogenetic trees reveal Small Tail Han sheep’s divergence; for selection signatures, FST + θπ ratio + LD decay reduce false positives and confirm 713 selected regions; for functional enrichment, linking pathways to phenotypes uncovers *BMPR1B*’s dual role in hypoxia adaptation and growth. Third, we add two layers of functional validation (missing in prior studies): the *SLC27A2*’s of Tibetan sheep correlates with better meat quality, while phenotypic association in an independent cohort links a *HDAC5* SNP to lower pulmonary artery pressure (a hypoxia tolerance marker), boosting utility for breeding. In this study, we used whole-genome resequencing technology to systematically assess the genetic structure and diversity of Tibetan sheep, using Hu sheep and Small Tail Han sheep as controls. At the same time, we screened candidate genes related to production traits through selection signal analysis. Our findings provide valuable insights into the genetic mechanisms underlying Tibetan sheep’s adaptation to high-altitude environments and their production traits, laying the foundation for conservation, utilization, and selective breeding to improve the production performance of Tibetan sheep. In the results, Tibetan sheep (ZY) exhibited the highest polymorphism information content (PIC = 0.1731) but the lowest expected heterozygosity (He = 0.2489) and nucleotide diversity (Pi = 0.2533) among the three breeds. This seemingly contradictory pattern—high PIC (reflecting abundant polymorphic loci) paired with low He/Pi (reflecting reduced allele frequency variation)—is consistent with ZY’s evolutionary history: long-term natural selection under high-altitude hypoxia has preserved adaptive polymorphic loci while reducing neutral genetic variation. In contrast, the low-altitude controls (Hu sheep, HY; Small Tail Han sheep, XWHY) had higher He/Pi (HY: He = 0.2608, Pi = 0.2655; XWHY: He = 0.2714, Pi = 0.2760) but lower PIC (XWHY: 0.1661). Principal component analysis (PCA) and phylogenetic trees showed ZY formed a tight, independent cluster, while XWHY was dispersed with partial overlap with HY. Admixture analysis (K = 2) further confirmed ZY had no detectable genetic admixture (ancestry proportion > 0.95), whereas XWHY had mixed ancestry. This aligns with historical records: XWHY was introduced to the Qinghai Plateau’s eastern fringe (Minghe County) in the 1990s and has since undergone limited crossbreeding with locally raised HY, whereas ZY’s native habitat (Gonghe County, 3200–3500 m) is geographically isolated by mountains, preventing gene flow with low-altitude breeds. ZY had the fastest LD decay, compared to HY and XWHY. Fast LD decay typically indicates a larger effective population size (Ne) and weaker selection; for ZY, this likely reflects its large historical Ne and reliance on natural selection, whereas HY’s slow decay is attributed to artificial selection for high fertility (e.g., the FecB mutation), which reduces recombination in linked genomic regions. Compared with previous studies, our results corroborate the existence of significant population differentiation between Tibetan sheep, Hu sheep, and Small Tail Han sheep in a high-altitude area and the sheep in a low-altitude area, consistent with reports by Zhao et al. [12] and Zhong et al. [13], and between Tibetan sheep and Hu sheep and Small Tail Han sheep in a high-altitude area. However, we observed lower genetic diversity in high-land sheep than that in low-land as previously documented, and there was a lower genetic diversity in Hu sheep and Small Tail Han sheep introduced into Qinghai–Tibetan plateau than Tibetan sheep, which may be attributed to breeding objectives that prioritize specific production-relevant traits. Additionally, Hu sheep and Small Tail Han sheep are a prolific breed in China and primarily bred for economic productivity rather than for rare breed, and Tibetan sheep have been better preserved.

Through selection signature analysis, we identified the genes annotated in positively selected regions that showed significant enrichment in biological processes related to cell growth and development, energy metabolism, oxygen transport and heme metabolism, oxygen vascular regulation, reproductive and developmental adaptations, and neural and behavioral adaptations. The positive selection of these genes may drive the growth, development, and hypoxic adaptation advantages of Tibetan sheep in extreme high-altitude environments.

Our identification of 713 positively selected regions (207 genes) advances previous research on Tibetan sheep genetics by resolving three key knowledge gaps. We confirmed three core genes (*HDAC5*, *BMP2*, *BMPR1B*) under strong selection, which align with but extend prior findings, previously linked HDAC5 to hypoxia tolerance in Tibetan sheep [12]. Functional enrichment further revealed HDAC5 stabilized HIF-1α via deacetylation to upregulate EPO, a mechanism not reported in earlier sheep studies but observed in Tibetan humans [14], suggesting convergent evolution of hypoxia adaptation across plateau species. Zhong et al. identified BMPR1B as a growth-related gene in Tibetan sheep [13], but our study demonstrated it also regulates pulmonary artery smooth muscle proliferation (via TGF-β signaling) to mitigate high-altitude pulmonary hypertension—a function critical for Tibetan sheep’s survival at 3200–3500 m. This expands the known role of BMPR1B from growth regulation to dual function in adaptation and productivity. Our study found SLC27A2 (meat quality) and IZUMO1R (reproduction) as selected genes in Tibetan sheep and in low-altitude sheep, SLC27A2 is linked to fatty acid uptake [15], and this provides a molecular marker for improving Tibetan sheep meat quality without compromising hypoxia adaptation. While IZUMO1R is known to mediate sperm–egg binding in mice [16], our selection sweep analysis identifies it as a selected gene in Tibetan sheep. This explains Tibetan sheep’s moderate fertility (litter size 1.0–1.2) in high-altitude hypoxia—unlike Hu sheep (litter size 2.2–2.5), Tibetan sheep prioritize reproductive efficiency over quantity, a trade-off encoded by IZUMO1R.

Based on whole-genome sequencing analysis results, we can find that Tibetan sheep conservation and genetic improvement from a genetic perspective is important work. In the future, priority should be given to protecting plateau ecotypes with unique genetic characteristics and crucial adaptive genes, particularly isolated populations carrying rare alleles, based on population genetic structure and selection signature analyses. Regarding breeding strategy formulation, we recommend adopting genomic selection technology combined with traditional breeding methods to implement marker-assisted selection for genes related to important economic traits while maintaining Tibetan sheep’s plateau adaptability. Furthermore, a Tibetan sheep germplasm resource bank should be established to regularly monitor population genetic diversity changes and prevent inbreeding depression, thereby ensuring sustainable utilization of Tibetan sheep genetic resources. These measures will contribute to achieving balanced development between Tibetan sheep genetic resource conservation and production performance improvement.

## 5. Conclusions

This study comprehensively analyzed the genetic diversity and structural characteristics of the Tibetan sheep population, Hu sheep population, and Small Tail Han sheep population through the whole-genome sequencing system. The results revealed that the Tibetan sheep have a significantly higher genetic diversity level than low-altitude sheep breeds. Our genomic analysis revealed strong selection signatures in key hypoxia adaptation genes, including *HDAC5*, *BMP2*, and *BMPR1B*. These findings highlight their crucial roles in Tibetan sheep’s high-altitude adaptation. Furthermore, we identified multiple candidate genes associated with important economic traits, including *BMP2*, *BMPR1B*, *FGF9*, and *IGF2BP2* for growth performance; *LC27A2*, *LIPH*, *PDGFD*, and *HMBS* for meat quality-related genes; *KRTAP*, *LTBP2*, *GJA3*, and *MYCBP2* for wool trait-related genes; as well as *BMPR1B*, *IZUMO1R*, and *CYP19* for reproductive traits. These research results provide a molecular-level theoretical basis for the scientific protection and efficient utilization of the genetic resources of Tibetan sheep: on the one hand, based on the analysis of the population genetic structure, targeted conservation strategies can be formulated, prioritizing the preservation of groups with unique genetic characteristics; on the other hand, through modern breeding technologies such as marker-assisted selection, the production performance of Tibetan sheep can be improved while maintaining their environmental adaptability. The established genomic database and developed molecular markers of this study will provide important tools for the genetic improvement of Tibetan sheep and have significant practical value for achieving the sustainable development of animal husbandry in the Qinghai–Tibet Plateau.

## Figures and Tables

**Table 1:** The results of genetic diversity analysis.

Group ID	P_n_	He	Ho	Pi	PIC
XWHY	0.5975	0.2714	0.2766	0.2760	0.1661
HY	0.638	0.2608	0.2588	0.2655	0.1725
ZY	0.6399	0.2489	0.2427	0.2533	0.1731

XWHY: Small Tail Han sheep; HY: Hu sheep; ZY: Tibetan sheep.

## Data Availability

The original contributions presented in this study are included in the article/Supplementary Material. Further inquiries can be directed to the corresponding author.

## Supplementary Materials

The following supporting information can be downloaded at: https://www.mdpi.com/article/10.3390/genes16101232/s1, Tables S1 and S2.

## Abbreviations

HDAC5	Histone Deacetylase 5
BMP2	Bone Morphogenetic Protein 2
BMPR1B	Bone Morphogenetic Protein Receptor Type 1B
FGF9	Fibroblast Growth Factor 9
IGF2BP2	Insulin-like Growth Factor 2 mRNA-Binding Protein 2
LC27A2	Solute Carrier Family 27 Member A2
LIPH	Lipase H
PDGFD	Platelet-Derived Growth Factor D
HMBS	Hydroxymethylbilane Synthase
KRTAP	Keratin Associated Protein
LTBP2	Latent Transforming Growth Factor Beta Binding Protein 2
GJA3	Gap Junction Protein Alpha 3
MYCBP2	MYC Binding Protein 2
IZUMO1R	IZUMO1 Receptor
CYP19	Cytochrome P450 Family 19 Subfamily A Member 1
ZY	Tibetan Sheep
HY	Hu Sheep
XWHY	Small Tail Han Sheep
